# Long-term causes of death in 888,003 ischemic stroke patients in Thailand: a nationwide retrospective study with 18-year follow-up

**DOI:** 10.1080/07853890.2026.2706870

**Published:** 2026-07-27

**Authors:** Prapassara Sirikarn, Pattarachanok Pukthaisong, Pipat Pattanapipitpaisal, Nisa Vorasoot, Narongrit Kasemsap, Somsak Tiamkao, Kannikar Kongbunkiat

**Affiliations:** ^a^ Department of Epidemiology and Biostatistics, Faculty of Public Health, Khon Kaen University, Khon Kaen, Thailand; ^b^ Integrated Epilepsy Research Group, Khon Kaen University, Khon Kaen, Thailand; ^c^ Brain Research and Innovation in Neuroscience, Khon Kaen University, Khon Kaen, Thailand; ^d^ Division of Neurology, Department of Medicine, Faculty of Medicine, Khon Kaen University, Khon Kaen, Thailand; ^e^ Northeastern Stroke Research Group, Khon Kaen University, Khon Kaen, Thailand

**Keywords:** Stroke, case fatality rate, post-discharge death

## Abstract

**Background:**

Ischemic stroke (IS) remains a major cause of mortality and disability worldwide, although the causes of death after stroke have changed. Long-term mortality data from low- and middle-income countries are limited. We investigated the fatality rates and causes of death in patients with IS in Thailand within and beyond 30 days post-discharge.

**Methods:**

We conducted a retrospective descriptive study using national hospital database records from 2004–2022. Patients with IS (ICD-10 code I63) were included without age or sex restrictions, with death status followed through 2022. The causes of death included stroke, complications, comorbidities, accidents, suicide, and unknown causes. Mortality was analyzed during hospitalization and ≤30 and >30 days post-discharge.

**Results:**

Among 888,003 patients with IS, the overall mortality was 47.6%: in-hospital 6.4%; ≤30 days post-discharge 8.2%; and >30 days post-discharge 33.1%. Mortality rates increased notably with age, exceeding 80% in those aged > 85 years. Stroke caused most in-hospital (58.6%) and short-term deaths (29.1%), whereas long-term mortality was primarily due to complications (30.7%) and comorbidities (30.5%). The leading comorbid causes were heart disease, dementia, and neoplasm; while pneumonia and sepsis were the primary fatal complications. Age-related debility, COVID-19 infection, and cardiovascular collapse were other major causes.

**Conclusion:**

This nationwide study revealed dynamic changes in post-IS causes, with stroke predominating early deaths, while complications and comorbidities determined long-term outcomes. These findings highlight the need for integrated acute and post-discharge stroke care strategies targeting infection prevention, comorbidity management, and elder care.

## Introduction

Stroke is the second leading cause of death and the third leading cause of disability worldwide [1,2]. Its burden encompasses both mortality and disability-adjusted life-years (DALYs), which account for both premature death and years lived with disability. Ischemic stroke (IS) is the predominant subtype (62.4% of cases in 2019), followed by intracerebral hemorrhage (27.9%) and subarachnoid hemorrhage (9.7%) [2].

Despite improvements in age-standardized rates, global IS deaths have increased. IS-related deaths increased from 2.04 million in 1990 to 3.29 million in 2019, with projections suggesting a further increase to 4.90 million by 2030 [3]. High-income countries exhibit declining age-standardized incidence and mortality rates, whereas low- and middle-income countries continue to bear a rising burden owing to limited healthcare resources and increased exposure to risk factors [4]. East Asia, Central Asia, and Sub-Saharan Africa have experienced particularly significant increases in obesity rates, driven by urbanization and lifestyle changes [5,6].

The causes of death in patients with IS vary depending on the time since stroke. In the early phase (≤1 month), stroke itself is the leading cause of death, accounting for approximately 57% of all deaths [7]. However, in the long term (>1 month to 5 years), non-vascular causes, such as cancer, become more recognizable, particularly in patients without a history of cancer [7,8]. Complications are significant contributors to mortality in patients with IS. For example, pneumonia accounts for 26.2% of deaths in the first 90 days after endovascular thrombectomy, whereas intracranial hemorrhage is responsible for 17.3% of deaths in the same period [9,10].

Despite IS being a significant burden, comprehensive data on evolving mortality rates over time, particularly in low- and middle-income countries, are lacking. Existing studies have limitations, including being restricted to specific populations (e.g. hospitalized patients or limited centers) [11,12] or focusing on short-term outcomes (30 days to 1 year) without considering long-term mortality and the causes of death [13,14]. While high-income countries document mortality shifts from stroke-related to comorbidities or medical complications [7–9], no large-scale studies have characterized these patterns in Southeast Asian populations, where healthcare systems, stroke etiology, and post-discharge care differ substantially from Western cohorts [15–18]. Thailand’s universal health coverage system, which covers 70% of the national population aged > 18 years, provides a unique opportunity to examine these mortality patterns in a middle-income Asian setting with different healthcare infrastructure and stroke risk factors compared to Western populations [19]. Thus, this study characterized the evolution of mortality among patients with IS in Thailand across three periods: hospitalization, short-term (≤30 days), and long-term (>30 days), providing essential data for developing time-specific interventions to reduce the long-term mortality burden in middle-income Southeast Asian settings.

## Materials and methods

### Study population

This was a retrospective descriptive study using data from the Thai Universal Coverage Scheme database from October 1, 2003 to September 30, 2022 (fiscal years 2004–2022), with vital status followed through September 30, 2022. The inclusion criteria were patients diagnosed with IS, identified by ICD-10 code I63, without age or sex restrictions. Patients with data errors were excluded: those with date inconsistencies (death recorded before discharge) and those with invalid discharge status codes. All remaining patients had complete data for essential variables.

### Data quality and diagnostic accuracy

Diagnostic accuracy for acute IS in Thailand is supported by reimbursement policies under the Universal Coverage Scheme, which typically require neuroimaging confirmation, most commonly brain CT. Because reimbursement is linked to diagnostic coding, clinicians and coding personnel are expected to ensure accurate documentation. Coding is performed by trained staff and subject to administrative audits, which may further enhance reliability.

### Outcome

The primary outcomes were mortality rates and causes of death, categorized as in-hospital death (during hospitalization), short-term death (within 30 days post-discharge), and long-term death (beyond 30 days post-discharge). The causes of death were classified into six groups: stroke, complications, comorbidities, accident, suicide, and unknown.

### Statistical analysis

Continuous data are reported as mean and standard deviation (SD), and categorical data are presented as frequencies and percentages. Mortality rates and causes of death were reported as percentages with 95% confidence intervals (95% CI). Confidence intervals for proportions were calculated using the Wald method. For suicide and accidental deaths, which were rare outcomes, Wilson score confidence intervals were calculated instead, as the Wald method has been shown to produce inadequate coverage probability when proportions approach zero [20]. Statistical analyses were performed using STATA software (version 18; StataCorp LLC, College Station, TX, USA). Data analysis was performed between October and November 2024.

### Sensitivity analysis

To assess the potential impact of the COVID-19 pandemic on mortality patterns, a sensitivity analysis was performed by excluding patients who died during the pandemic period (2020–2022). This approach allows examination of long-term mortality patterns in the absence of COVID-19 as a contributing cause of death.

## Results

### Characteristics of participants

**Figure 1.**
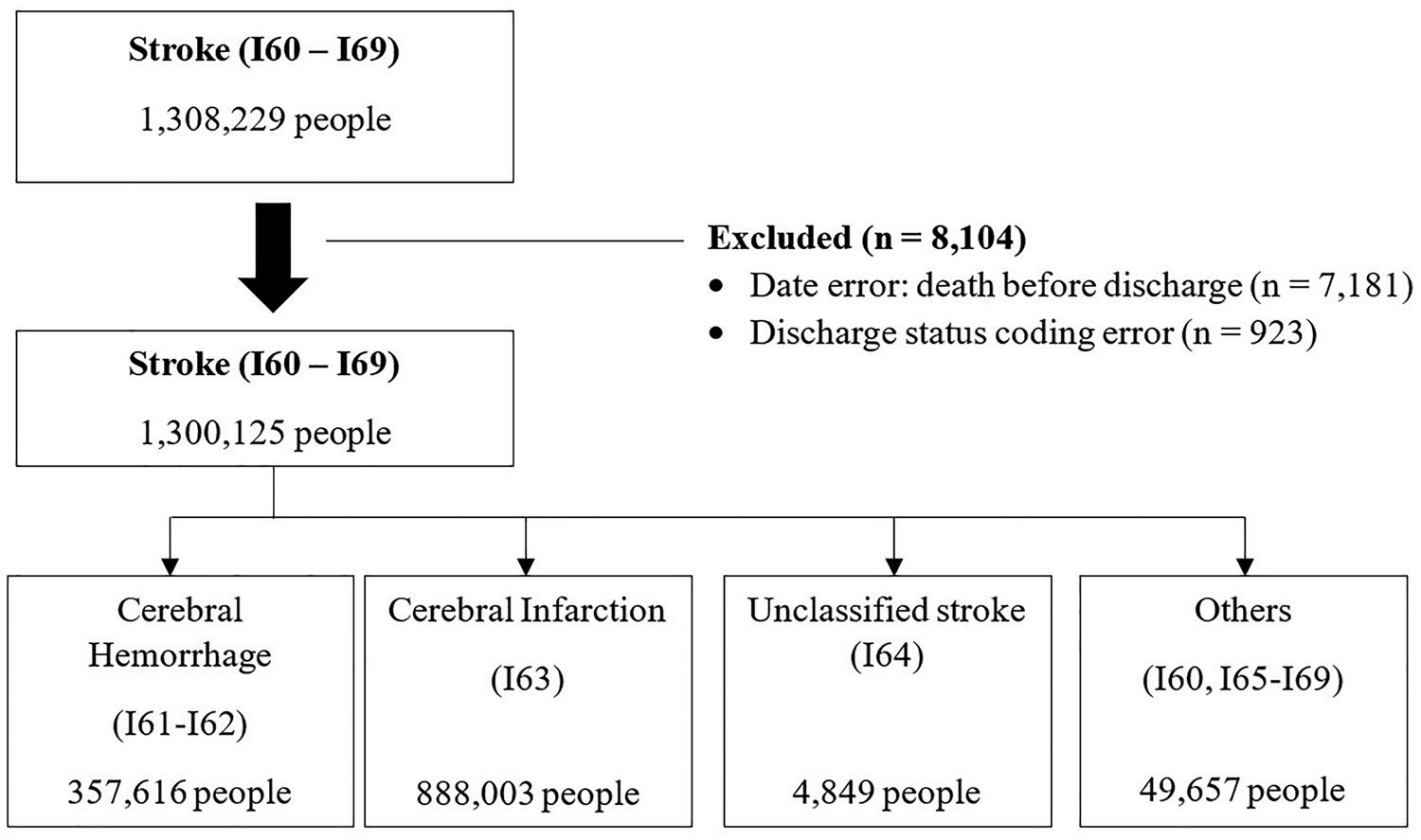
Patient selection flowchart and classification of stroke subtypes based on ICD-10 codes (I60–I69).

We initially identified 1,308,229 patients with stroke (ICD-10 codes I60–I69). After excluding 7,181 patients due to date errors (death before discharge) and 923 patients due to discharge status coding errors, 1,300,125 patients remained for analysis. Among these, 357,616 patients (27.5%) were diagnosed with cerebral hemorrhage (I61–I62), 888,003 patients (68.3%) with cerebral infarction (I63), 4,849 patients (0.4%) with unclassified stroke (I64), and 49,657 patients (3.8%) with other stroke-related diagnoses (I60, I65–I69) (Figure 1).

The baseline characteristics of the 888,003 patients with IS are summarized in Table 1. The cohort comprised 53.8% males and 46.2% females. The mean age at first admission was 65 years (SD = 13.56), with the majority of patients falling within the 56–65 (25.0%) and 66–75 (27.5%) age ranges. Hypertension was the most common comorbidity (54.3%), followed by diabetes mellitus (27.4%) and chronic kidney disease (7.5%).

**Table 1.** Baseline characteristics of patients with ischemic stroke (*n* = 888,003).

Characteristics	*n*	%
Sex		
Male	477,747	53.8%
Female	410,256	46.2%
Age at first admission (years)		
<18	2,193	0.3%
18–25	3,149	0.3%
26–35	12,633	1.4%
36–45	50,537	5.7%
46–55	136,423	15.4%
56–65	221,869	25.0%
66–75	244,479	27.5%
76–85	171,949	19.4%
> 85	44,771	5.0%
Mean (SD)	65.29	(13.56)
Median (Min:Max)	66	(0:122)
Comorbidity		
Hypertension (I10-I13)	481,959	54.3%
Diabetes mellitus (E10-E14)	243,068	27.4%
Chronic kidney disease (N18)	66,410	7.5%
Pulmonary disease (J44, J45, J93, J85, I270)	25,414	2.9%
Hyperlipidemia (E87.5)	14,832	1.7%
Valvular heart disease (I05-I09)	10,127	1.1%
Neoplasm (C00-C97)	10,107	1.1%
Dementia (G30, G31)	9,216	1.0%
Atrial fibrillation (I48.9)	8,162	0.9%
Cirrhosis of liver (K74)	3,697	0.4%
HIV infection (B24)	3,298	0.4%
Obesity (E66)	1,966	0.2%

Abbreviations: HIV, human immunodeficiency virus; SD, standard deviation.

### Mortality rates in patients with is

Among 888,003 patients diagnosed with IS, the overall mortality rate was 47.6% (95%CI: 47.5–47.7). In-hospital death occurred in 6.4% (95%CI: 6.3–6.4) of patients, while short-term and long-term death rates were 8.2% (95%CI: 8.1–8.2) and 33.1% (95%CI: 32.9–33.2), respectively. The mortality rate increased progressively with age. Patients under 18 years had a total mortality rate of 25.7% compared with 81.4% among those aged > 85 years. In-hospital death rates ranged from 3.7% in the 18–25 age group to 12.6% in those over 85 years. Similarly, short-term death rates ranged from 3.0 to 19.3%, and long-term death rates ranged from 11.4 to 49.5%, demonstrating a clear age-dependent increase in mortality among patients with IS (Table 2).

**Table 2.** Mortality rates in patients with ischemic stroke.

Age at admission	*n*	Total			In-hospital death			Short-term death			Long-term death		
		*n*	%	95%Cl	*n*	%	95%CI	*n*	%	95%CI	*n*	%	95%CI
<18	2,209	568	25.7	23.9 to 27.5	111	5.0	4.1 to 5.9	123	5.6	4.6 to 6.5	334	15.1	13.6 to 16.6
18–25	3,158	573	18.1	16.8 to 19.5	117	3.7	3.1 to 4.4	96	3.0	2.4 to 3.6	360	11.4	10.3 to 12.51
26–35	12,745	2,902	22.8	22.0 to 23.5	606	4.8	4.4 to 5.1	504	4.0	3.6 to 4.3	1,792	14.1	13.5 to 14.7
36–45	51,080	12,332	24.1	23.8 to 24.5	2,365	4.6	4.5 to 4.8	1,966	3.8	3.7 to 4.0	8,001	15.7	15.4 to 15.9
46–55	137,279	38,614	28.1	27.9 to 28.4	6,109	4.5	4.3 to 4.6	5,647	4.1	4.0 to 4.2	26,858	19.6	19.4 to 19.8
56–65	221,904	82,224	37.1	36.9 to 37.3	10,978	4.9	4.9 to 5.0	12,043	5.4	5.3 to 5.5	59,203	26.7	26.5 to 26.9
66–75	243,774	129,536	53.1	52.9 to 53.3	15,607	6.4	6.3 to 6.5	20,959	8.6	8.5 to 8.7	92,970	38.1	37.9 to 38.3
76–85	171,268	119,950	70.0	69.8 to 70.3	15,078	8.8	8.7 to 8.9	22,643	13.2	13.1 to 13.4	82,229	48.0	47.8 to 48.3
> 85	44,586	36,306	81.4	81.1 to 81.8	5,626	12.6	12.3 to 12.9	8,599	19.3	18.9 to 19.7	22,081	49.5	49.1 to 49.9
**Overall**	**888,003**	**423,005**	**47.6**	**47.5 to 47.7**	**56,597**	**6.4**	**6.3 to 6.4**	**72,580**	**8.2**	**8.1 to 8.2**	**293,828**	**33.1**	**32.9 to 33.2**

Abbreviation: CI, confidence interval.

### Causes of death in patients with is

Building on the analysis of mortality rates, we further dissected the data to identify the primary causes of death among the study population, revealing insightful trends that among the 423,005 patients with IS who died between 2004 and 2022, the leading causes of death were complications (28.4%, 95%CI: 28.3–28.6), comorbidities (25.4%, 95%CI: 25.3–25.5), and stroke (21.5%, 95%CI: 21.4–21.7). Among in-hospital deaths (*n* = 56,597), the most common cause was stroke (58.6%), followed by complications (27.1%) and comorbidities (6.6%). In short-term deaths (*n* = 72,580), stroke remained the most common cause (29.1%), with complications (20.1%) and comorbidities (19.3%) as the next most frequent. In contrast, for long-term deaths (*n* = 293,828), complications (30.7%) and comorbidities (30.5%) were the leading causes, with stroke contributing to a smaller proportion (12.5%). These findings highlight the dynamic shifts in the causes of death over time after IS (Table 3).

**Table 3.** Causes of death in patients with ischemic stroke.

Cause of death	Total (*n* = 423,005)			In-hospital death (*n* = 56,597)			Short-term death (*n* = 72,580)			Long-term death (*n* = 293,828)		
	*n*	%	95%CI	*n*	%	95%CI	*n*	%	95%CI	*n*	%	95%CI
1. Stroke	91,137	21.5	21.4 to 21.7	**33,167**	**58.6**	**58.2 to 59.0**	**21,095**	**29.1**	**28.7 to 29.4**	36,875	12.5	12.4 to 12.7
2. Comorbidity	107,416	25.4	25.3 to 25.5	3,751	6.6	6.4 to 6.8	14,017	19.3	19.0 to 19.6	89,648	30.5	30.3 to 30.7
3. Complication	**120,199**	**28.4**	**28.3 to 28.6**	15,357	27.1	26.8 to 27.5	14,580	20.1	19.8 to 20.4	**90,262**	**30.7**	**30.6 to 30.9**
4. Accident	4,015	0.9	0.9 to 1.0	117	0.2	0.2 to 0.2	316	0.4	0.4 to 0.5	3,582	1.2	1.2 to 1.3
5. Suicide	1,519	0.4	0.3 to 0.4	9	0.02	0.01 to 0.03	81	0.1	0.1 to 0.1	1,429	0.5	0.5 to 0.5
6. Others	66,783	15.8	15.7 to 15.9	486	0.9	0.8 to 0.9	12,694	17.5	17.2 to 17.8	53,603	18.2	18.1 to 18.4
7. Unknown	31,936	7.6	7.5 to 7.6	3,710	6.6	6.4 to 6.8	9,797	13.5	13.2 to 13.7	18,429	6.3	6.2 to 6.4

Note: Bold values indicate the cause category with the highest percentage within each time period.

Abbreviation: CI, confidence interval.

### Causes of death due to complications

Complications were the primary cause of death in patients with IS. The principal causes of death among 120,199 patients with IS who died from complications were pneumonia (27.6%), septicemia (26.0%), and heart failure (15.6%). In-hospital deaths (*n* = 15,357) were predominantly due to septicemia (35.9%) and pneumonia (32.0%), while heart failure accounted for 6.1% of the deaths. For short-term deaths (*n* = 14,580), pneumonia (29.2%) predominated, followed by septicemia (22.8%) and heart failure (17.7%). In patients who died in the long term (*n* = 90,262), pneumonia (26.6%) and septicemia (24.8%) predominated, whereas heart failure accounted for 16.9% of deaths. Other notable causes included acute renal failure (6.3%), respiratory failure (5.5%), and multiple organ failure (2.6%) (Table 4).

**Table 4.** Causes of death due to complications in patients with ischemic stroke.

Cause of death	Total (*n* = 120,199)		In-hospital death (*n* = 15,357)		Short-term death (*n* = 14,580)		Long-term death (*n* = 90,262)	
	*n*	%	*n*	%	*n*	%	*n*	%
Pneumonia	**33,169**	**27.6**	**4,919**	**32.0**	**4,261**	**29.2**	**23,989**	**26.6**
Septicemia	**31,199**	**26.0**	**5,516**	**35.9**	**3,326**	**22.8**	**22,357**	**24.8**
Heart failure	**18,760**	**15.6**	930	6.1	**2,576**	**17.7**	**15,254**	**16.9**
Acute renal failure	7,566	6.3	266	1.7	1,162	8.0	6,138	6.8
Respiratory failure	6,641	5.5	**1,187**	**7.7**	830	5.7	4,624	5.1
Multiple organ failure	3,159	2.6	339	2.2	326	2.2	2,494	2.8
Urinary tract infection	3,143	2.6	278	1.8	305	2.1	2,560	2.8
Shock	2,443	2.0	295	1.9	273	1.9	1,875	2.1
Gastrointestinal haemorrhage	2,047	1.7	135	0.9	193	1.3	1,719	1.9
Decubitus ulcer and pressure area	1,339	1.1	24	0.2	56	0.4	1,259	1.4
Meningitis	1,092	0.9	141	0.9	305	2.1	646	0.7
Pulmonary edema	1,028	0.9	77	0.5	84	0.6	867	1.0
Infection on specified	995	0.8	37	0.2	37	0.2	921	1.0
Metabolic acidosis - alkalosis	763	0.6	91	0.6	83	0.6	589	0.7
Cerebral edema	686	0.6	439	2.9	170	1.2	77	0.1
Others	6,169	5.1	683	4.4	593	4.1	4,893	5.4

Note: Bold values indicate the top three leading causes of death from complications within each time period.

### Causes of death due to comorbidities

Comorbidities were the second leading cause of death among IS patients. The principal causes of death among 107,416 patients with IS who died from comorbidities were heart disease (22.6%), dementia (20.1%), and neoplasm (17.9%). However, the distribution of the causes varied significantly by period. Heart disease was the predominant cause of in-hospital deaths (*n* = 3,751), heart disease was the predominant cause (56.9%), followed by neoplasms (10.7%) and hypertension (8.5%). Notably, dementia contributed to only 1.2% of all deaths. In contrast, both short-term deaths (*n* = 14,017) and long-term deaths (*n* = 89,648) showed a different pattern, with dementia (21.7% and 20.7%, respectively) and neoplasms (13.4% and 18.9%, respectively) emerging as major contributors, alongside heart disease. Other notable comorbidities included diabetes mellitus (12.6%), hypertension (9.6%), and chronic kidney disease (6.9%) (Table 5).

**Table 5.** Causes of death due to comorbidities in patients with ischemic stroke.

Cause of death	Total (*n* = 107,416)		In-hospital death (*n* = 3,751)		Short-term death (*n* = 14,017)		Long-term death (*n* = 89,648)	
	*n*	%	*n*	%	*n*	%	*n*	%
Heart disease	**24,291**	**22.6**	**2,135**	**56.9**	**2,858**	**20.4**	**19,298**	**21.5**
Dementia	**21,644**	**20.1**	45	1.2	**3,039**	**21.7**	**18,560**	**20.7**
Neoplasm	**19,266**	**17.9**	**401**	**10.7**	1,877	13.4	**16,988**	**18.9**
Diabetes mellitus	13,579	12.6	143	3.8	**2,271**	**16.2**	11,165	12.4
Hypertension	10,274	9.6	**320**	**8.5**	1,943	13.9	8,011	8.9
Chronic kidney disease	7,449	6.9	242	6.5	636	4.5	6,571	7.3
Pulmonary tuberculosis	1,690	1.6	95	2.5	245	1.7	1,350	1.5
Cirrhosis of liver	1,604	1.5	67	1.8	200	1.4	1,337	1.5
Emphysematous of lung	1,407	1.3	48	1.3	171	1.2	1,188	1.3
HIV/AIDS	577	0.5	43	1.1	77	0.5	457	0.5
Epilepsy	468	0.4	39	1.0	47	0.3	382	0.4
Hyperlipidemia	215	0.2	6	0.2	54	0.4	155	0.2
Hepatic failure	213	0.2	14	0.4	30	0.2	169	0.2
Anemia	197	0.2	7	0.2	27	0.2	163	0.2
Hyperthyroidism	195	0.2	35	0.9	29	0.2	131	0.2
Others	4,347	4.1	111	3.0	513	3.7	3,723	4.2

Note: Bold values indicate the top three leading causes of death from comorbidities within each time period.

Abbreviations: AIDS, acquired immunodeficiency syndrome; HIV, human immunodeficiency virus.

### Causes of death due to other causes

“Others” was the fourth most common cause of death among patients with IS. The principal causes of death among the 66,783 patients with IS whose deaths were attributed to other causes, age-related physical debility (ICD-10: R54) predominated (87.6%). However, the distribution varied by period: in-hospital deaths (*n* = 486) were more split between age-related debility (20.8%) and COVID-19 (24.3%), while both short-term (*n* = 12,694) and long-term (*n* = 53,603) deaths were dominated by age-related physical debility (93.8 and 86.7%, respectively). COVID-19 accounted for 1.0% of short-term and 5.8% of long-term deaths. Cardiovascular collapse contributed to 1.7% of short-term and 1.3% of long-term deaths. Other causes included atherosclerotic vascular disease (0.6%), hypoxemia (0.6%), nutritional deficiency (0.5%), and sleep apnea (0.4%) (Table 6).

**Table 6.** Causes of death due to other causes in patients with ischemic stroke.

Causes of death	Total (*n* = 66,783)		In-hospital death (*n* = 486)		Short-term death (*n* = 12,694)		Long-term death (*n* = 53,603)	
	*n*	%	*n*	%	*n*	%	*n*	%
Age-related debility	**58,489**	**87.6**	**101**	**20.8**	**11,901**	**93.8**	**46,487**	**86.7**
Covid-19	**3,370**	**5.0**	**118**	**24.3**	**127**	**1.0**	**3,125**	**5.8**
Cardiovascular collapse	**905**	**1.4**	4	0.8	**220**	**1.7**	**681**	**1.3**
Atherosclerotic vascular disease	407	0.6	32	6.6	37	0.3	338	0.6
Hypoxemia	379	0.6	**55**	**11.3**	45	0.4	279	0.5
Nutritional deficiencies	306	0.5	5	1.0	20	0.2	281	0.5
Sleep apnea	268	0.4	0	0.0	42	0.3	226	0.4
Seizure	225	0.3	38	7.8	29	0.2	158	0.3
Volume overload	204	0.3	15	3.1	15	0.1	174	0.3
Bacterial foodborne intoxications	201	0.3	5	1.0	29	0.2	167	0.3
Abdominal aortic aneurysm, ruptured	188	0.3	4	0.8	6	0.1	178	0.3
Fever unspecified	159	0.2	3	0.6	40	0.3	116	0.2
Asphyxia	156	0.2	12	2.5	19	0.1	125	0.2
Acute bronchitis	145	0.2	5	1.0	12	0.1	128	0.2
Viral hepatitis	88	0.1	1	0.2	16	0.1	71	0.1
Others	1,293	1.9	88	18.1	136	1.1	1,069	2.0

Note: Bold values indicate the top three leading causes of death from other causes within each time period.

Abbreviations: COVID-19, coronavirus disease 2019.

### Sensitivity analysis

A sensitivity analysis excluding 115,709 patients (13.0%) who died during the pandemic period (2020–2022) was performed to examine mortality patterns in the absence of COVID-19 as a contributing cause of death. Of 772,294 patients included, 39.8% (95%CI: 39.7–39.9%) died. Mortality patterns and causes of death remained highly consistent with the main analysis. In-hospital mortality was 5.9% (vs 6.4% in main analysis), short-term mortality was 7.7% (vs 8.2%), and long-term mortality was 26.3% (vs 33.1%). The distribution of causes of death was nearly identical: complications 28.0% (vs 28.4%), comorbidities 24.1% (vs 25.4%), stroke 21.3% (vs 21.5%), and others 16.9% (vs 15.8%). Within each category, the ranking and relative proportions of specific causes remained unchanged (Supplementary Tables S1-S5).

## Discussion

In our nationwide study of 888,003 patients with IS in Thailand, the overall mortality rate was 47.6%, with 6.4% dying during hospitalization, 8.2% dying within 30 days post-discharge (short-term mortality), and 33.1% dying thereafter (long-term mortality). Mortality rates increase markedly with age, reaching over 80% in patients older than 85 years. The causes of death varied by period: stroke itself was the predominant cause of in-hospital and short-term mortality, whereas complications and comorbidities were the leading causes of long-term mortality.

### Baseline characteristics

The mean age was 65 years, with a male predominance (53.8%). Hypertension (54.3%) and diabetes mellitus (27.4%) were the most common comorbidities. Our cohort was younger than the North American (70 years) and European (68 years) cohorts [15,16], similar to the Singaporean cohort (67 years), but slightly older than the Indian cohort (62 years) [17,18]. Male predominance was consistent across regions, although it was lower than that in North America (61%) and Europe (58%) [15,16]. The prevalence of comorbidities was notably lower, including hypertension (54.3%) compared to North America (75%) and Asia (>80%) [18,21], and diabetes (27.4%) compared to Asian cohorts (∼40%) [17,18] and North American ones (37%) [15]. The relatively lower prevalence of certain comorbidities, such as diabetes, compared with reports from high-income countries may partly reflect demographic differences. Patients with IS in Thailand tend to present at a younger age, which may result in lower cumulative exposure to metabolic and cardiovascular comorbidities. Differences in population structure, healthcare access, and disease detection rates may also contribute to international variation in comorbidity prevalence. Beyond demographic factors, variations in lifestyle patterns (dietary habits, urbanization) and differences in study methodologies (hospital-based vs. population-based registries) may further contribute to the observed international discrepancies.

### Mortality rates in patients with is

Mortality rates were 6.4% in-hospital, 8.2% short-term, and 33.1% long-term, increasing progressively with age. Our in-hospital mortality rate was lower than that in Oman (16.4%) [22], Mexico (13.9% in public hospitals) [23], Denmark (14.2% at 30-days) [24], and the Czech Republic [25], likely reflecting advancements in acute IS management, including the widespread use of intravenous thrombolysis and mechanical thrombectomy during our study period [26,27]. Conversely, long-term mortality (33.1%) exceeded the 1-year mortality rate among US Medicare beneficiaries (23.8–26.5%) [28] and the 12-month mortality rate in Oman (25.4%) [22]. This higher rate reflects our extended longitudinal design, which followed patients for up to 18 years (2004–2022), allowing for the accumulation of mortality over time. The age-dependent mortality increase aligns with international studies showing that older age and comorbidity burden substantially influence both short- and long-term ischemic stroke outcomes [29,30]. Variations may also reflect differences in healthcare systems, post-stroke secondary prevention practices, and socioeconomic factors [31,32].

### Causes of death in patients with is

Complications were the leading cause of death (28.4%), followed by comorbidities (25.4%) and the direct effects of stroke (21.5%). This aligns with the findings of Wang et al. who reported that approximately 40–50% of deaths were attributable to stroke-related complications [33,34]. However, our comorbidity-related deaths (25.4%) exceeded prior reports of 10–30% [35,36], whereas direct stroke deaths (21.5%) were lower than those in previous reports (30–40%) [37]. These differences reflect our extended 18-year follow-up compared to shorter studies, improved acute ischemic stroke management during our enrollment period, and comprehensive case ascertainment. Our data revealed dynamic mortality patterns: stroke predominated early deaths, whereas complications and comorbidities became increasingly prominent in the long term. This evolution underscores the importance of ongoing surveillance for both acute complications and chronic comorbidities in survivors of ischemic stroke.

### Causes of death due to complications

Among patients with IS who died due to complications, pneumonia (27.6%) was the leading cause, followed by septicemia (26.0%), heart failure (15.6%), acute renal failure (6.3%), respiratory failure (5.5%), and multiple organ failures (2.6%). These findings align with previous reports showing pneumonia as the most common fatal post-stroke complication (3.9-15% incidence) [38–41] and sepsis as a significant contributor (1.7–48.4% prevalence) [40–42]. Our higher pneumonia and sepsis rates compared to those in high-income countries (2.7–3.8%) [43] underscore the need for enhanced prevention and management strategies. Key interventions should include systematic dysphagia screening, early mobilization, pneumonia-preventive stroke care bundles, infection surveillance, strict aseptic practices for catheter and ventilation management, and multidisciplinary rehabilitation. Strengthening post-acute care pathways and expanding access to comprehensive stroke units, particularly in resource-limited settings, are crucial for reducing fatal complications and improving patient outcomes.

### Causes of death due to comorbidities

Among patients with IS who died from comorbidities, heart disease was the leading cause (22.6%), consistent with prior studies showing strong cardiac-stroke associations [44–46]. Dementia (20.1%) and neoplasms (17.9%) followed, aligning with evidence that cognitive impairment worsens outcomes [47,48] and cancer-associated hypercoagulability increases immunosuppression and mortality [46]. Notably, hypertension (9.6%) and diabetes mellitus (12.6%) contributed less than previous studies reporting a 24–30% increased mortality risk [49–51]. Chronic kidney disease (6.9%) was also less frequent, despite prior evidence of a significant CKD-associated stroke mortality risk [52]. These differences may reflect variations in population characteristics, access to healthcare, stroke management protocols, risk factor control, and death attribution methodology, particularly given the long-term nationwide follow-up.

### Causes of death due to other causes

The leading causes of death among patients with IS who died from other causes were age-related debility (87.6%), COVID-19 (5.0%), and cardiovascular collapse (1.4%). Age-related debility, the most common cause, aligns with previous research showing that advanced age significantly impacts stroke mortality and functional outcomes [53,54]. The high COVID-19 mortality reflects global observations of substantially increased short-term mortality (10.9%-37.9%) among patients [55–57]. Cardiovascular collapse as the third most common cause aligns with previous findings that heart failure and acute cardiac events contribute to a poor prognosis [58,59]. These findings may reflect the population demographics, healthcare systems, post-stroke care quality, and impact of COVID-19. The similarities across studies identifying cardiovascular complications and aging as critical factors highlight the global challenges in managing vascular comorbidities, while the differences underscore the need for tailored strategies focusing on geriatric care, infection prevention, and cardiovascular management in Thai stroke survivors.

### Public health and policy implications

From a health-system perspective, reducing long-term post-stroke mortality in Thailand requires two key strategies: prevention of fatal complications and systematic management of comorbidities after discharge. As pneumonia and septicemia were leading fatal complications, implementation of standardized stroke infection-prevention bundles—such as early dysphagia screening, aspiration precautions, oral care, early mobilization, respiratory physiotherapy when indicated, catheter stewardship, and early sepsis recognition—should be prioritized. In addition, the predominance of heart disease, dementia, and malignancy as long-term causes of death supports structured post-discharge follow-up, including cardiovascular assessment, vascular risk optimization, cognitive screening, and guideline-based cancer screening. Integrating these measures into stroke-unit quality indicators and follow-up programs may improve sustainable long-term survival.

### Strengths and limitations

The strengths of this study include a large sample size and data covering approximately 70% of the national population, enhancing generalizability for public health planning. Time-based mortality rate analysis provides detailed insights into mortality patterns across hospitalization and post-discharge periods, aiding in the design of surveillance strategies. The extended 18-year follow-up enabled a comprehensive assessment of long-term mortality. Additionally, our sensitivity analysis excluding 115,709 pandemic-period deaths demonstrated highly consistent mortality patterns and cause-of-death distributions (all differences <2%), indicating that our findings represent stable long-term patterns independent of the COVID-19 pandemic’s influence.

However, this study has several limitations. First, selection bias is possible because this study used data from the Universal Coverage Scheme, which may not fully represent patients under other insurance schemes or in the private sector. Differences in socioeconomic status, rehabilitation access, and secondary prevention may affect long-term outcomes; thus, generalizability to non-UCS populations should be interpreted cautiously. Second, as this study was based on administrative ICD-10 coding without direct clinical or imaging validation, some degree of diagnostic misclassification cannot be excluded, though systematic requirements for neuroimaging confirmation under Universal Coverage Scheme reimbursement policies likely minimize this concern. Third, the analysis lacked data on stroke severity and detailed ischemic stroke subtype classification, potentially affecting mortality estimates. Fourth, treatment information (intravenous thrombolysis or thrombectomy) and functional outcomes (modified Rankin Scale and activities of daily living) were unavailable, which limited the interpretation of the outcomes. Fifth, sex-stratified analyses of mortality patterns and causes of death were not performed in this study. Although males comprised 53.8% of our cohort, previous literature suggests that biological, hormonal, and behavioral differences between sexes may influence stroke risk factor profiles, comorbidity burden, and long-term outcomes. Future sex-stratified analyses are warranted to better understand potential differences in mortality patterns and post-stroke complications in the Thai population. Finally, death certification accuracy was limited for out-of-hospital deaths, where causes were often attributed to age-related debility without physician confirmation, potentially affecting the reliability of short- and long-term mortality analyses.

## Conclusion

In this nationwide Thai study of patients with IS, we identified high mortality rates that increased with age. Stroke predominated early deaths, whereas complications and comorbidities drove long-term mortality. Our findings align with global patterns but reveal local differences, including a younger age and distinct comorbidity profiles. The predominance of heart disease, dementia, and pneumonia as causes of death, along with significant contributions from COVID-19 and cardiovascular collapse, underscores the need for integrated post-stroke care targeting both acute complications and chronic morbidities. These results emphasize the necessity of continuous improvement in acute care and long-term secondary prevention to reduce mortality and improve outcomes in stroke survivors.

## Supplementary Material

Supplementary.docx

## Data Availability

The data supporting the findings of this study are available upon request from the corresponding author. The data are not publicly available because of the need to protect the privacy of the research participants.
