# Supplementary material for: Long-term causes of death in 888,003 ischemic stroke patients in Thailand: a nationwide retrospective study with 18-year follow-up
Source: Ann Med. 2026 Jul 27;58(1):2706870. doi: 10.1080/07853890.2026.2706870 (PMC13410538; doi:10.1080/07853890.2026.2706870)
Supplement: Supplementary.docx [file IANN_A_2706870_SM4734.docx]

**Table S1** Sensitivity analysis of mortality rates in patients with ischemic stroke

| **Age at admission** | **n** | **Total** | | | **In-hospital death** | | | **Short-term death** | | | **Long-term death** | | |
| --- | --- | --- | --- | --- | --- | --- | --- | --- | --- | --- | --- | --- | --- |
|  |  | **n** | **%** | **95%Cl** | **n** | **%** | **95%CI** | **n** | **%** | **95%CI** | **n** | **%** | **95%CI** |
| < 18 | 2,156 | 515 | 23.9 | 22.1 to 25.7 | 102 | 4.7 | 3.8 to 5.6 | 114 | 5.3 | 4.3 to 6.2 | 299 | 13.9 | 12.4 to 15.3 |
| 18 – 25 | 3,027 | 442 | 14.6 | 13.3 to 15.9 | 95 | 3.1 | 2.5 to 3.8 | 88 | 2.9 | 2.3 to 3.5 | 259 | 8.6 | 7.6 to 9.6 |
| 26 – 35 | 12,034 | 2,191 | 18.2 | 17.5 to 18.9 | 491 | 4.1 | 3.7 to 4.4 | 424 | 3.5 | 3.2 to 3.9 | 1,276 | 10.6 | 10.1 to 11.2 |
| 36 – 45 | 47,797 | 9,049 | 18.9 | 18.6 to 19.3 | 1,946 | 4.1 | 3.9 to 4.2 | 1,675 | 3.5 | 3.3 to 3.7 | 5,428 | 11.4 | 11.1 to 11.6 |
| 46 – 55 | 126,122 | 27,457 | 21.8 | 21.5 to 22.0 | 4,872 | 3.9 | 3.8 to 4.0 | 4,665 | 3.7 | 3.6 to 3.8 | 17,920 | 14.2 | 14.0 to 14.4 |
| 56 – 65 | 197,681 | 58,001 | 29.3 | 29.1 to 29.5 | 8,709 | 4.4 | 4.3 to 4.5 | 9,725 | 4.9 | 4.8 to 5.0 | 39,567 | 20.0 | 19.8 to 20.2 |
| 66 – 75 | 208,755 | 94,517 | 45.3 | 45.1 to 45.5 | 12,435 | 6.0 | 5.9 to 6.1 | 17,251 | 8.3 | 8.1 to 8.4 | 64,831 | 31.1 | 30.9 to 31.3 |
| 76 – 85 | 140,203 | 88,885 | 63.4 | 63.1 to 63.6 | 12,188 | 8.7 | 8.5 to 8.8 | 18,469 | 13.2 | 13.0 to 13.4 | 58,228 | 41.5 | 41.3 to 41.8 |
| > 85 | 34,519 | 26,239 | 76.0 | 75.6 to 76.5 | 4,337 | 12.6 | 12.2 to 12.9 | 6,722 | 19.5 | 19.1 to 19.9 | 15,180 | 44.0 | 43.5 to 44.5 |
| **Overall** | **772,294** | **307,296** | **39.8** | **39.7 to 39.9** | **45,175** | **5.9** | **5.8 to 5.9** | **59,133** | **7.7** | **7.6 to 7.7** | **202,988** | **26.3** | **26.2 to 26.4** |

Abbreviations: CI, confidence interval.

**Table S2** Sensitivity analysis of causes of death in patients with ischemic stroke

| **Cause of death** | **Total**  **(n = 307,296)** | | | **In-hospital death**  **(n =45,175)** | | | **Short-term death**  **(n = 59,133)** | | | **Long-term death**  **(n = 202,988)** | | |
| --- | --- | --- | --- | --- | --- | --- | --- | --- | --- | --- | --- | --- |
|  | **n** | **%** | **95%CI** | **n** | **%** | **95%CI** | **n** | **%** | **95%CI** | **n** | **%** | **95%CI** |
| 1. Stroke | 65,576 | 21.3 | 21.2 to 21.5 | **25,604** | **56.7** | **56.2 to 57.1** | **15,642** | **26.5** | **26.1 to 26.8** | 24,330 | 12.0 | 11.8 to 12.1 |
| 2. Comorbidity | 73,919 | 24.1 | 23.9 to 24.2 | 3,045 | 6.7 | 6.5 to 7.0 | 11,070 | 18.7 | 18.4 to 19.0 | 59,804 | 29.5 | 29.3 to 29.7 |
| 3. Complication | **86,100** | **28.0** | **27.9 to 28.2** | 12,438 | 27.5 | 27.1 to 27.9 | 11,385 | 19.3 | 18.9 to 19.6 | **62,277** | **30.7** | **30.5 to 30.9** |
| 4. Accident | 2,715 | 0.9 | 0.9 to 0.9 | 89 | 0.2 | 0.2 to 0.2 | 247 | 0.4 | 0.4 to 0.5 | 2,379 | 1.2 | 1.1 to 1.2 |
| 5. Suicide | 1,039 | 0.3 | 0.3 to 0.4 | 6 | 0.01 | 0.01 to 0.03 | 53 | 0.1 | 0.1 to 0.1 | 980 | 0.5 | 0.5 to 0.5 |
| 6. Others | 52,027 | 16.9 | 16.8 to 17.1 | 293 | 0.7 | 0.6 to 0.7 | 11,487 | 19.4 | 19.1 to 19.7 | 40,247 | 19.8 | 19.7 to 20.0 |
| 7. Unknown | 25,920 | 8.4 | 8.3 to 8.5 | 3,700 | 8.2 | 7.9 to 8.4 | 9,249 | 15.6 | 15.3 to 15.9 | 12,971 | 6.4 | 6.3 to 6.5 |

Note: Bold values indicate the cause category with the highest percentage within each time period.

Abbreviations: CI, confidence interval.

**Table S3** Sensitivity analysis of causes of death due to complications in patients with ischemic stroke

| **Cause of death** | **Total**  **(n = 86,100)** | | **In-hospital death**  **(n = 12,438)** | | **Short-term death**  **(n = 11,385)** | | **Long-term death**  **(n = 62,277)** | |
| --- | --- | --- | --- | --- | --- | --- | --- | --- |
|  | **n** | **%** | **n** | **%** | **n** | **%** | **n** | **%** |
| Pneumonia | **23,391** | **27.2** | **3,896** | **31.3** | **3,213** | **28.2** | **16,282** | **26.1** |
| Septicemia | **23,322** | **27.1** | **4,604** | **37.0** | **2,623** | **23.0** | **16,095** | **25.8** |
| Heart failure | **13,677** | **15.9** | 795 | 6.4 | **2,164** | **19.0** | **10,718** | **17.2** |
| Acute renal failure | 5,362 | 6.2 | 230 | 1.8 | 911 | 8.0 | 4,221 | 6.8 |
| Respiratory failure | 4,822 | 5.6 | **932** | **7.5** | 668 | 5.9 | 3,222 | 5.2 |
| Multiple organ failure | 2,330 | 2.7 | 293 | 2.4 | 269 | 2.4 | 1,768 | 2.8 |
| Urinary tract infection | 1,905 | 2.2 | 196 | 1.6 | 189 | 1.7 | 1,520 | 2.4 |
| Shock | 1,753 | 2.0 | 248 | 2.0 | 221 | 1.9 | 1,284 | 2.1 |
| Gastrointestinal haemorrhage | 1,424 | 1.7 | 98 | 0.8 | 142 | 1.2 | 1,184 | 1.9 |
| Decubitus ulcer and pressure area | 949 | 1.1 | 17 | 0.1 | 40 | 0.4 | 892 | 1.4 |
| Meningitis | 804 | 0.9 | 121 | 1.0 | 246 | 2.2 | 437 | 0.7 |
| Infection on specified | 650 | 0.8 | 29 | 0.2 | 28 | 0.2 | 593 | 1.0 |
| Pulmonary edema | 638 | 0.7 | 55 | 0.4 | 57 | 0.5 | 526 | 0.8 |
| Cerebral edema | 539 | 0.6 | 337 | 2.7 | 137 | 1.2 | 65 | 0.1 |
| Metabolic acidosis - alkalosis | 426 | 0.5 | 60 | 0.5 | 55 | 0.5 | 311 | 0.5 |
| Others | 4,108 | 4.8 | 527 | 4.2 | 422 | 3.7 | 3,159 | 5.1 |

Note: Bold values indicate the top three leading causes of death from complications within each time period.

**Table S4** Sensitivity analysis of causes of death due to comorbidities in patients with ischemic stroke

| **Cause of death** | **Total**  **(n = 73,919)** | | **In-hospital death**  **(n = 3,045)** | | **Short-term death**  **(n = 11,070)** | | **Long-term death**  **(n = 59,804)** | |
| --- | --- | --- | --- | --- | --- | --- | --- | --- |
|  | **n** | **%** | **n** | **%** | **n** | **%** | **n** | **%** |
| Heart disease | **17,249** | **23.3** | **1,727** | **56.7** | **2,305** | **20.8** | **13,217** | **22.1** |
| Dementia | **14,072** | **19.0** | 31 | 1.0 | **2,233** | **20.2** | **11,808** | **19.7** |
| Neoplasm | **13,085** | **17.7** | **297** | **9.8** | 1,438 | 13.0 | **11,350** | **19.0** |
| Diabetes milletus | 9,779 | 13.2 | 128 | 4.2 | **1,909** | **17.2** | 7,742 | 12.9 |
| Hypertension | 7,204 | 9.7 | **281** | **9.2** | 1,577 | 14.3 | 5,346 | 8.9 |
| Chronic kidney disease | 4,534 | 6.1 | 191 | 6.3 | 444 | 4.0 | 3,899 | 6.5 |
| Pulmonary tuberculosis | 1,246 | 1.7 | 74 | 2.4 | 200 | 1.8 | 972 | 1.6 |
| Cirrhosis of liver | 1,160 | 1.6 | 60 | 2.0 | 155 | 1.4 | 945 | 1.6 |
| Emphysematous of lung | 1,124 | 1.5 | 44 | 1.4 | 157 | 1.4 | 923 | 1.5 |
| HIV/AIDS | 462 | 0.6 | 36 | 1.2 | 66 | 0.6 | 360 | 0.6 |
| Epilepsy | 339 | 0.5 | 32 | 1.1 | 43 | 0.4 | 264 | 0.4 |
| Hyperlipidemia | 162 | 0.2 | 5 | 0.2 | 46 | 0.4 | 111 | 0.2 |
| Hepatic failure | 144 | 0.2 | 10 | 0.3 | 22 | 0.2 | 112 | 0.2 |
| Hyperthyroidism | 144 | 0.2 | 32 | 1.1 | 23 | 0.2 | 89 | 0.1 |
| Anemia | 124 | 0.2 | 5 | 0.2 | 22 | 0.2 | 97 | 0.2 |
| Others | 3,091 | 4.2 | 92 | 3.0 | 430 | 3.9 | 2,569 | 4.3 |

Note: Bold values indicate the top three leading causes of death from comorbidities within each time period.

Abbreviations: AIDS, acquired immunodeficiency syndrome; HIV, human immunodeficiency virus.

**Table S5** Sensitivity analysis of causes of death due to other causes in patients with ischemic stroke

| **Causes of death** | **Total**  **(n = 52,027)** | | **In-hospital death**  **(n = 293)** | | **Short-term death**  **(n = 11,487)** | | **Long-term death**  **(n = 40,247)** | |
| --- | --- | --- | --- | --- | --- | --- | --- | --- |
|  | **n** | **%** | **n** | **%** | **n** | **%** | **n** | **%** |
| Age-related debility | **48,510** | **93.2** | **98** | **33.4** | **10,931** | **95.2** | **37,481** | **93.1** |
| Cardiovascular collapse | **814** | **1.6** | 4 | 1.4 | **214** | **1.9** | **596** | **1.5** |
| Atherosclerotic vascular disease | 283 | 0.5 | 24 | 8.2 | 26 | 0.2 | 233 | 0.6 |
| Hypoxemia | 201 | 0.4 | **33** | **11.3** | 26 | 0.2 | 142 | 0.4 |
| Nutritional deficiencies | 203 | 0.4 | 4 | 1.4 | 14 | 0.1 | 185 | 0.5 |
| Sleep apnea | 199 | 0.4 | 0 | 0.0 | 31 | 0.3 | 168 | 0.4 |
| Seizure | 153 | 0.3 | 32 | 10.9 | 17 | 0.1 | 104 | 0.3 |
| Volume overload | 120 | 0.2 | 14 | 4.8 | 9 | 0.1 | 97 | 0.2 |
| Bacterial foodborne intoxications | 149 | 0.3 | 4 | 1.4 | 27 | 0.2 | 118 | 0.3 |
| Abdominal aortic aneurysm, ruptured | 122 | 0.2 | 3 | 1.0 | 3 | 0.03 | 116 | 0.3 |
| Fever unspecified | 144 | 0.3 | 3 | 1.0 | 39 | 0.3 | 102 | 0.3 |
| Asphyxia | 102 | 0.2 | 9 | 3.1 | 14 | 0.1 | 79 | 0.2 |
| Acute bronchitis | 84 | 0.2 | 4 | 1.4 | 7 | 0.1 | 73 | 0.2 |
| Viral hepatitis | 66 | 0.1 | 0 | 0.0 | 14 | 0.1 | 52 | 0.1 |
| Others | 877 | 1.7 | 61 | 20.8 | 115 | 1.0 | 701 | 1.7 |

Note: Bold values indicate the top three leading causes of death from other causes within each time period.

Abbreviations: COVID-19, coronavirus disease 2019.
